# Multimodal EEG and explainable machine learning characterize neuroticism-related neurodynamic heterogeneity in mild cognitive impairment during visuospatial working memory

**DOI:** 10.3389/fnagi.2026.1889176

**Published:** 2026-07-27

**Authors:** Wang Wei, Zhao Ziwei, Liu Yong, Hou Junlin, Qin Zhongpeng, Han Heyun, Chen Zhuo, Wang Jubo, Song Peiling, Zhan Xianghong

**Affiliations:** 1 Department of Basic Theory of Traditional Chinese Medicine, Traditional Chinese Medicine (Zhong Jing) School, Henan University of Chinese Medicine, Zhengzhou, China; 2 Zhengzhou Key Laboratory for Chinese and Western Integrative Prevention and Treatment of Brain Cognitive Disease, Zhengzhou, China; 3 Henan Province Engineering Technology Research Center for Chinese and Western Integrative Prevention and Treatment of Brain Cognitive Disease, Zhengzhou, China; 4 Research and Experimental Center, Traditional Chinese Medicine (Zhong Jing) School, Henan University of Chinese Medicine, Zhengzhou, China; 5 Department of Physiology, School of Medicine, Henan University of Chinese Medicine, Zhengzhou, China; 6 Department of Basic Teaching for Integrated Traditional Chinese and Western Medicine, Traditional Chinese Medicine (Zhong Jing) School, Henan University of Chinese Medicine, Zhengzhou, China; 7 Department of Rehabilitation, Henan Province Hospital of Traditional Chinese Medicine (The Second Affiliated Hospital of Henan University of Chinese Medicine), Zhengzhou, China; 8 Department of Integrated Traditional Chinese and Western Medicine, Traditional Chinese Medicine (Zhong Jing) School, Henan University of Chinese Medicine, Zhengzhou, China

**Keywords:** electroencephalography, event-related potentials, explainable machine learning, functional connectivity, mild cognitive impairment, neuroticism, phase-amplitude coupling, visuospatial working memory

## Abstract

**Background:**

As a personality trait associated with emotional instability and negative emotional tendencies, neuroticism is considered to be closely related to the risk of cognitive decline in old age. However, the neurodynamic abnormalities associated with elevated neuroticism among individuals with MCI remain unclear.

**Methods:**

Cognitive screening based in the community was performed in Zhumadian, Henan Province, and Xingtai, Hebei Province, China. Based on converted scores on the Neuroticism subscale of the Eysenck Personality Questionnaire, individuals with MCI were categorized into high-neuroticism MCI (HN-MCI) and non-elevated neuroticism MCI (NEN-MCI) groups. After EEG recruitment, behavioral-task completion, EEG quality control, and frequency matching by sex, age, and years of education, 53 participants with HN-MCI and 53 with NEN-MCI were included. All participants engaged in a modified visuospatial working memory test during the recording of 64-channel EEG data. Behavioral measures, event-related potentials, time-frequency power, phase-amplitude coupling, and dwPLI-based functional connectivity features were extracted. A stacking ensemble learning model integrated with SHAP-based interpretability analysis was employed for multimodal feature fusion and classification.

**Results:**

In comparison to the NEN-MCI group, the HN-MCI group exhibited lower Montreal Cognitive Assessment (MoCA) total and visuospatial/executive scores, prolonged reaction times, and elevated inverse efficiency scores. EEG analyses indicated increased P2 and P3 amplitudes, reduced N2 amplitude, increased theta activity, enhanced theta-to-low-alpha phase-amplitude coupling, and reinforced theta-band functional connectivity in the HN-MCI group. The stacking model differentiated the two groups with an AUC of 0.858, and permutation testing indicated that model performance was significantly above chance (permutation *p* < 0.001). SHAP analysis identified parietal P3 amplitude as the most influential feature.

**Conclusion:**

Individuals with HN-MCI exhibit multilevel neurodynamic abnormalities across temporal, spectral, cross-frequency, and network domains during visuospatial working memory processing, accompanied by impaired visuospatial/executive function and reduced behavioral efficiency. Neuroticism-related emotional traits may contribute to a sustained high-load and low-efficiency neural processing pattern in individuals with MCI. Multimodal EEG combined with explainable machine learning provides a useful framework for characterizing personality-related neurodynamic heterogeneity in MCI.

## Introduction

Mild cognitive impairment (MCI) is widely regarded as an intermediate stage between normal aging and dementia (Geda, 2012; Petersen et al., 2014). Longitudinal studies have shown that some individuals with MCI remain cognitively stable over time (stable MCI, sMCI), some progress to dementia (progressive MCI, pMCI), and a considerable proportion may return to normal cognition under appropriate intervention (reversible MCI, rMCI) (Gao J. et al., 2025). This marked clinical heterogeneity suggests that individuals with MCI may have distinct underlying neuropathological mechanisms. Identifying key factors that shape MCI heterogeneity has therefore become an important issue in cognitive neuroscience and aging research.

Neuroticism, a personality trait associated with emotional instability, heightened stress reactivity, and a tendency toward negative affect, has been linked to the risk of cognitive decline, executive dysfunction, and dementia outcomes in older adults (Boyle et al., 2010; Manning et al., 2017; Widiger, 2017; Yoneda et al., 2023; Gao et al., 2025; Shen et al., 2025). Neuroticism may participate in cognitive aging and disease progression and may further influence cognitive and neurophysiological differences among individuals with MCI. From a neurobiological perspective, higher levels of neuroticism are closely associated with chronic activation of the hypothalamic–pituitary–adrenal (HPA) axis (McEwen and Gianaros, 2011; Montoliu et al., 2020). This may subject the prefrontal cortex and hippocampus, areas abundant in glucocorticoid receptors, to sustained elevations in glucocorticoids, consequently compromising synaptic plasticity and neural network integrity, and ultimately heightening the risk of cognitive decline (Buchanan and Loveday, 2018; Montoliu et al., 2020; Yoneda et al., 2023; Ariño-Braña et al., 2025). Neuroticism is also associated with functional abnormalities in the amygdala-prefrontal network, which may reduce emotion regulation efficiency and executive control (Servaas et al., 2013; Herman et al., 2020). Thus, neuroticism may not only increase the risk of cognitive decline, but also shape the neural processing patterns of individuals with MCI.

Working memory is one of the core cognitive components that is vulnerable in the early stage of MCI. Working memory-related EEG markers can sensitively reflect changes in cognitive load in individuals with MCI and distinguish MCI subtypes with different disease trajectories (Missonnier et al., 2007). Elevated neuroticism may interfere with the maintenance, selection, and updating of task-relevant information by depleting attentional control resources (Eysenck et al., 2007; Osaka et al., 2013; Moya et al., 2026). Since visuospatial working memory places particularly high demands on attentional control and executive resources, which are functions known to be affected by neuroticism, it may provide a sensitive task window for detecting neuroticism-related cognitive load effects in MCI. Furthermore, visuospatial working memory has been regarded as a preliminary indicator of the transition from Mild Cognitive Impairment to Alzheimer’s Disease (Lou et al., 2015; Seo et al., 2021), hence reinforcing its significance in this context.

Although previous studies have linked neuroticism to cognitive decline and working-memory inefficiency, it remains unclear at which levels of neural processing elevated neuroticism alters visuospatial working-memory dynamics in individuals with MCI. The present study therefore focused on whether HN-MCI is characterized by a multilevel neurodynamic pattern rather than by a single behavioral or electrophysiological abnormality. Because neuroticism-related cognitive inefficiency may involve overt performance, stimulus-locked processing, oscillatory resource allocation, cross-frequency coordination, and interregional network synchronization, a multimodal EEG framework was used to capture complementary aspects of task processing.

**Figure 1:**
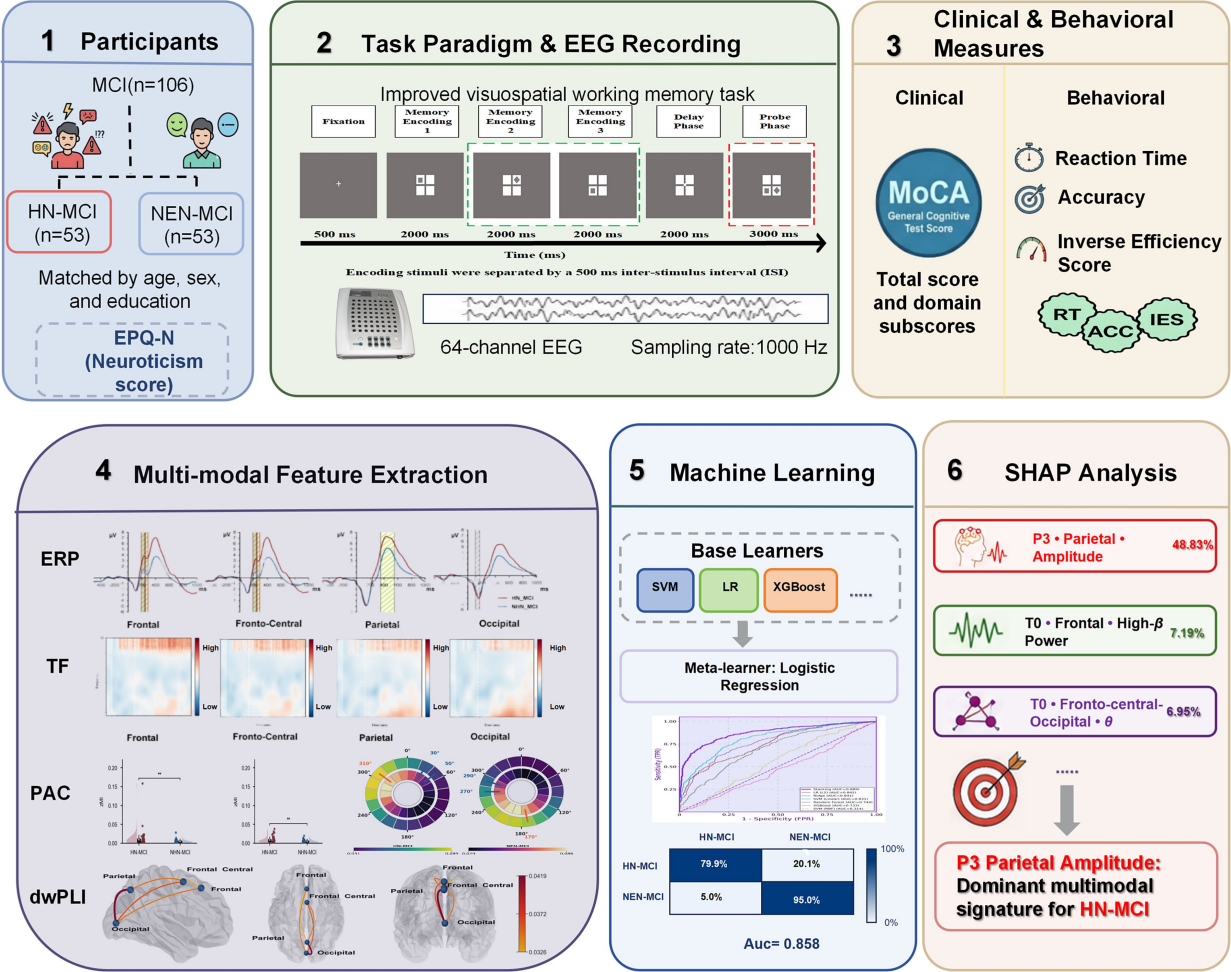
Overview of the study design and analytical pipeline.

Each modality was selected to address a specific level of this research question. Behavioral indices, including accuracy, reaction time, and inverse efficiency score, were used to quantify overt task performance and processing efficiency (Bruyer and Brysbaert, 2011). ERPs were used to characterize the temporal sequence of attentional allocation, stimulus evaluation, conflict monitoring, and working-memory updating (Luck et al., 2000; Luck, 2005). Time-frequency power was used to examine task-related oscillatory activity associated with cognitive load, attentional control, and working-memory maintenance (Chaddad et al., 2023; van Engen et al., 2024; Wischnewski et al., 2024). PAC was included to assess cross-frequency coordination between slower control-related rhythms and faster local neural activity (Dvorak and Fenton, 2014; Mariscal et al., 2021). dwPLI-based functional connectivity was used to evaluate phase-lagged interregional synchronization while reducing the influence of volume conduction (Smith et al., 2022). A stacking ensemble model was then constructed for multimodal feature fusion, and SHAP-based interpretability analysis was applied to identify the features that contributed most to HN-MCI and NEN-MCI classification (Sylvester et al., 2024). This analytical strategy is consistent with recent work emphasizing EEG-derived biomarkers, multimodal machine learning, stability-driven EEG connectivity feature selection, and explainable artificial intelligence in neuropsychiatric and cognitive research (Naeim and Narimani, 2026a, 2026b; Naeim et al., 2026; Narimani and Naeim, 2025; Mikaeili et al., 2025). We hypothesized that individuals with HN-MCI would exhibit distinct abnormalities across behavioral, temporal, spectral, cross-frequency, and network levels, reflecting a high-load and low-efficiency neural processing pattern during visuospatial working memory (see Figure 1).

## Materials and methods

### Participants

Community-based cognitive screening was conducted between June 2024 and September 2025 in Zhumadian, Henan Province, and Xingtai, Hebei Province, China. A total of 2,086 questionnaires were distributed, and 1,951 valid questionnaires were returned. During community-based screening, trained assessors collected medical history and self-reported health information using a structured community screening form. This form included an item asking whether participants currently had or had previously had major medical, neurological, or psychiatric conditions, including a history of psychiatric disorders such as depression or anxiety disorders (Supplementary Figure S1). Participants with known or clearly reported psychiatric disorders, neurological disorders, dementia, or other severe conditions that could substantially affect cognitive function were excluded from the eligible MCI sample. According to the diagnostic criteria for MCI, 858 individuals with MCI were identified. Among them, 118 participants were excluded because their converted scores on the Lie subscale of the Eysenck Personality Questionnaire (EPQ-L) were ≥70, indicating insufficient response validity. The remaining 740 eligible MCI participants included 237 individuals with HN-MCI and 503 with NEN-MCI.

**Figure 2:**
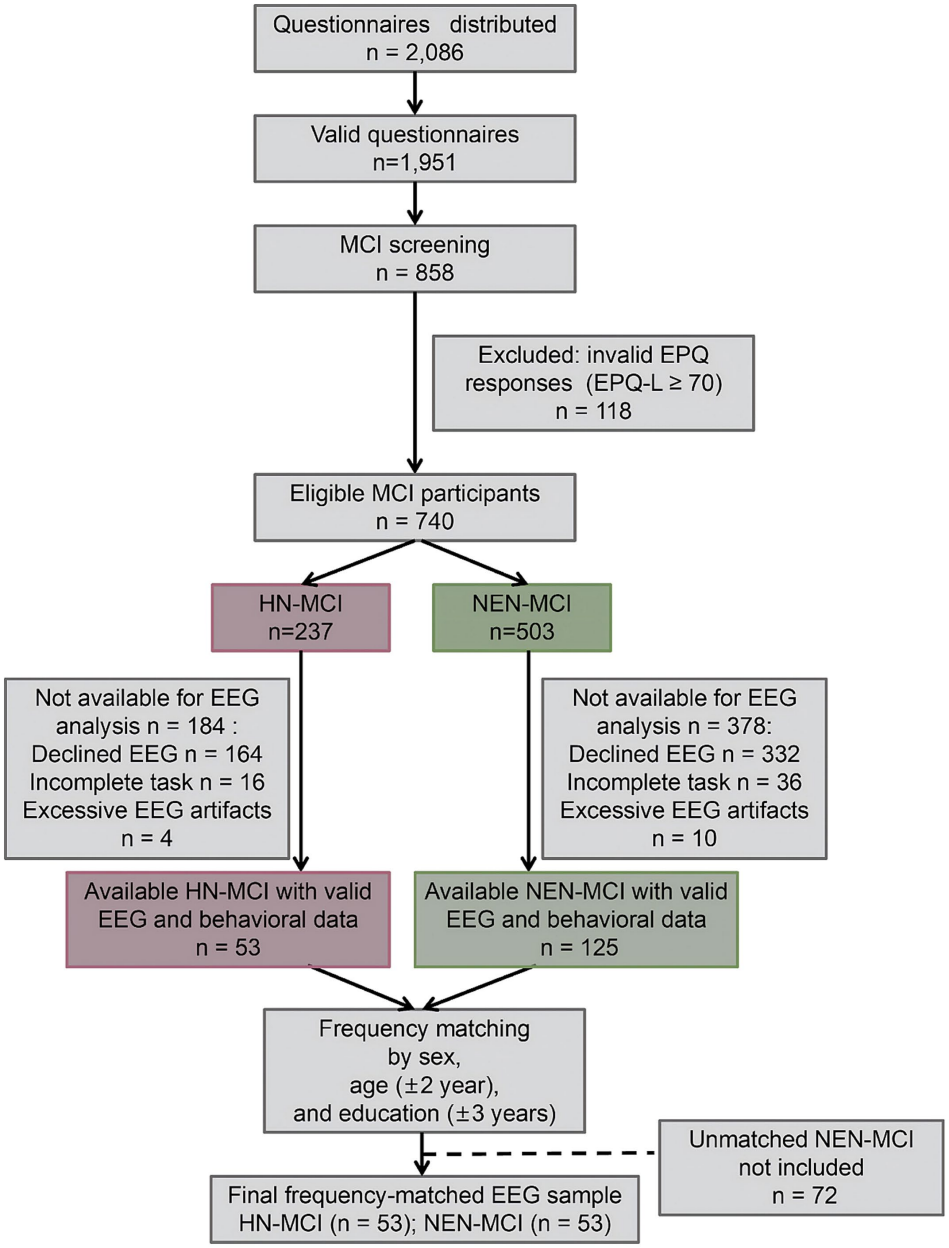
Flowchart of participant recruitment, screening, and matching procedures.

Participants were classified according to converted scores on the Neuroticism subscale of the Eysenck Personality Questionnaire (EPQ-N). The converted EPQ scores are standardized T scores based on Chinese normative data. According to the Chinese EPQ scoring system, scores >61.5 represent the typical high range of the corresponding personality dimension. Therefore, EPQ-N converted scores >61.5 were used to define high-neuroticism MCI (HN-MCI), whereas scores ≤61.5 were defined as non-elevated neuroticism MCI (NEN-MCI). This threshold was used for normative personality-trait stratification and should not be interpreted as a clinical diagnostic cut-off (Gong, 1984; Wang et al., 2022). After EEG recruitment, completion of the behavioral paradigm, and EEG quality control, valid EEG and behavioral data were available for 53 HN-MCI participants and 125 NEN-MCI participants. Because the available HN-MCI sample determined the maximum size of a balanced group comparison, all 53 HN-MCI participants with valid EEG and behavioral data were retained. To minimize demographic confounding and group-size imbalance, 53 NEN-MCI participants were selected from the 125 available NEN-MCI participants using frequency matching by sex, age (±2 years), and years of education (±3 years). The final frequency-matched EEG sample comprised 106 participants, with 53 participants in each group. To further evaluate potential selection bias related to EEG recruitment and data availability, we compared the available demographic, EPQ-N, and cognitive variables between EEG-included and EEG-not-included eligible MCI participants within each neuroticism group, including sex, age, education, EPQ-N, MoCA total score, and MoCA visuospatial/executive score (Supplementary Table S1; Figure 2).

### Diagnostic criteria for MCI

The diagnostic criteria for MCI were as follows (Petersen et al., 2001; Portet et al., 2006; Petersen et al., 2018):(1) The participant or a family member reported the presence of cognitive complaints, with a Global Deterioration Scale (GDS) score of 2–3; (2) the participant or an informant reported a decline in cognitive function over the past year, indicating a change from a previously higher level of performance; (3) trained assessors administered the Beijing version of the Montreal Cognitive Assessment (MoCA-BJ) according to standardized procedures (Lu et al., 2011; Tian et al., 2020), and the corrected total MoCA score (adjusted for years of education where applicable) was >18 and <26; and (4) activities of daily living were largely preserved, with an Activity of Daily Living (ADL) scale score <26 (Lawton and Brody, 1969). Cognitive impairment that did not reach the level of dementia was determined comprehensively based on GDS grade (≤3), MoCA score, and ADL score <26.

The exclusion criteria included: (1) inability or unwillingness to participate in the survey or assessment; (2) dementia, neurological disorders, severe psychiatric or psychological conditions, such as depression, anxiety disorders, and schizophrenia, or significant organ diseases that may impair cognitive function. All participants furnished written informed consent. The Ethics Committee of the First Affiliated Hospital of Henan University of Chinese Medicine approved the study protocol (approval number. 2023HL-400-01).

### Clinical assessments

All volunteers aged 45–65 years, encompassing middle-aged and older adults, completed cognitive and personality evaluations conducted by uniformly trained assessors using standardized protocols. The Beijing version of the Montreal Cognitive Assessment (MoCA-BJ) was used to assess global cognitive function. This measure encompasses various cognitive areas, including attention, concentration, executive function, memory, language, visuospatial skills, abstract reasoning, computation, and orientation. The revised MoCA total score and domain scores were incorporated into subsequent studies.

### Task paradigm

This study employed a modified sequential presentation-delayed judgment visuospatial working memory task to mitigate the potential floor effect observed in persons with mild cognitive impairment (MCI) and older adults during traditional high-load N-back tasks (Belleville et al., 2007). The task preserved the fundamental framework of sequence encoding, delay maintenance, and probe assessment, while adjusting the task load to align with the cognitive capacity of the target demographic to stimulate visuospatial encoding, maintenance, and inhibitory control mechanisms.

**Figure 3:**
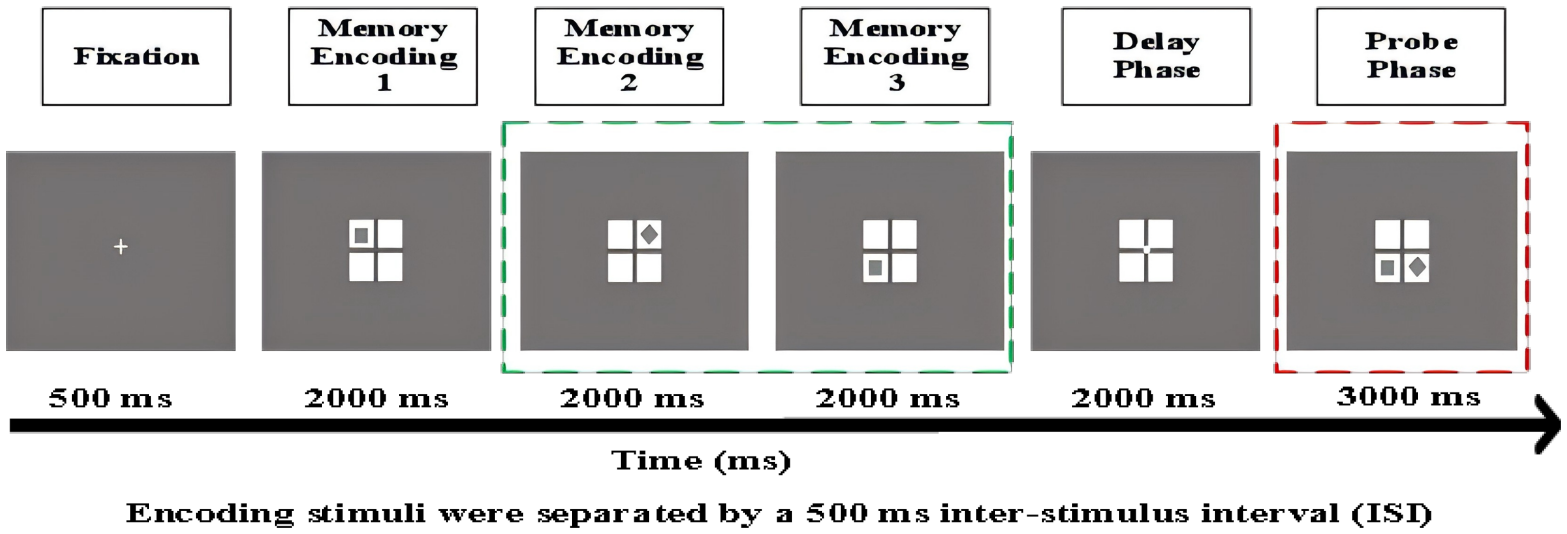
Flowchart of the improved visuospatial working memory task.

The experimental stimuli were gray squares and diamonds arranged in a 2 × 2 grid against a white background. Each trial comprised four phases: fixation, sequence encoding, delay maintenance, and probing judgment. At the beginning of each trial, a fixation point was presented for 500 ms, followed by three visuospatial stimuli presented sequentially. Each stimulus lasted 2,000 ms, with an interstimulus interval of 500 ms. After sequence presentation, a 2,000-ms delay maintenance phase occurred. Two probe figures were then presented simultaneously, and participants were asked to judge whether the probes fully matched the last two stimuli in the encoding sequence in both location and shape. Participants pressed the F key for matching trials and the J key for nonmatching trials. The maximum response time was 3,000 ms. The formal experiment consisted of two blocks, with 60 trials in each block (Figure 3).

### EEG acquisition and preprocessing

EEG and behavioral data were recorded simultaneously using a NeuroScan NuAmps 64-channel EEG system. Participants wore a 64-channel Ag/AgCl electrode cap, with electrodes placed according to the international extended 10–20 system. EEG signals were recorded with the bilateral mastoid electrodes M1 and M2 as references. The sampling rate was 1,000 Hz, and electrode impedance was kept below 5 kOhm. Behavioral measures included accuracy (ACC), reaction time (RT), and inverse efficiency score (IES). The IES was used to integrate the speed-accuracy trade-off (Bruyer and Brysbaert, 2011; Castellan Jr and Restle, 2014) and was calculated according to Equation 1, where ACC denotes the proportion of correct responses.


$$\mathrm{IES}=\frac{\mathrm{RT}}{\mathrm{ACC}}$$ (1)

EEG preprocessing was performed using the MNE-Python toolbox in Python (v3.13.5). A 0.1–30 Hz band-pass filter was applied, and a 48–52 Hz notch filter was used to remove power-line noise. Epochs were extracted from 500 ms before to 1,000 ms after stimulus onset, and baseline correction was performed using the mean activity during the 500-ms prestimulus period. Continuous data were first visually inspected, and segments with obvious artifacts were removed. Bad channels were repaired by interpolation from neighboring channels when necessary. Independent component analysis (ICA) was used to identify and remove non-neural artifacts, including ocular and muscular artifacts (Delorme et al., 2007). Epochs with amplitudes exceeding ±80 μV were rejected. After artifact rejection, the remaining valid trials for each participant were retained for subsequent analyses.

### EEG feature extraction

#### Regions of interest

Based on previous literature and the typical scalp distribution of ERP components, a hypothesis-driven approach was adopted. Twelve core electrodes from the international 10–20 system were divided into four anatomical regions of interest (ROIs): frontal (F3, Fz, F4), fronto-central (FC3, FCz, FC4), parietal (P3, Pz, P4), and occipital (PO7, Oz, PO8) regions (Picton et al., 2000; Luck, 2005). This ROI definition was kept strictly consistent across all feature modalities.

#### ERP analysis

For ERP time-domain analysis, four core ERP components were extracted within specific ROIs. Time windows and scalp distributions were determined according to the grand-average waveforms, topographic maps, and previous studies. N1 (110–170 ms) was extracted from occipital electrodes; P2 (190–250 ms) and N2 (240–290 ms) were extracted from frontal and fronto-central electrodes; and P3 (350–550 ms) was extracted from parietal electrodes. Mean amplitude and peak latency were extracted within the corresponding time window for each component. Amplitude was quantified as the mean potential within the time window rather than as a single-point peak value; therefore, the extracted amplitude values could be positive or negative.

#### Time-frequency analysis

For time-frequency (TF) analysis, Morlet wavelet transformation was applied to the preprocessed EEG signals to characterize dynamic modulation of neural oscillations across different cognitive stages (Herrmann et al., 2004). The number of wavelet cycles was adaptively adjusted as a function of frequency (n_cycles = f/2, minimum = 3), and the frequency range was set to 1–30 Hz. To reduce interindividual variability and improve the comparability of evoked oscillatory components, baseline correction was performed using dB transformation with the 500-ms prestimulus interval as the baseline. The dB-transformed power was calculated according to Equation 2.


$${\text{Power}}_{\mathrm{dB}}=10\times \log_{10}(\frac{{\text{Power}}_{\text{active}}}{{\text{Power}}_{\text{baseline}\_\text{mean}}})$$ (2)

According to the task processing stages and classical oscillatory frequency bands, the TF results were further mapped onto a three-dimensional analytical framework of ROI x frequency band x time window. The spatial dimension used the four aforementioned ROIs. The frequency spectrum encompassed theta (4–8 Hz), low alpha (8–10 Hz), high alpha (10–13 Hz), low beta (13–20 Hz), and high beta (20–30 Hz). The temporal dimension encompassed the N1, P2, N2, P3, and T0 (0–1,000 ms) intervals. This method facilitated the concurrent characterization of local oscillatory alterations at various cognitive stages and the maintenance of neuronal activity throughout the entire task period.

#### Phase-amplitude coupling analysis

Phase-amplitude coupling (PAC) was quantified using the modulation index (MI). Low-frequency phase and high-frequency amplitude envelopes were extracted separately using the Hilbert transform. The low-frequency phase was divided into 18 equally spaced phase bins, and the mean high-frequency amplitude corresponding to each phase bin was calculated. A null distribution was then constructed using 200 time-shift surrogate iterations, and the raw MI was transformed into a standardized zMI value (Dvorak and Fenton, 2014; Scherer et al., 2022). MI was calculated according to Equation 3, where H(P) denotes the Shannon entropy of the mean amplitude distribution and Hmax = ln(N). To reduce noise effects, MI values were further converted into z scores (zMI).


$$\mathrm{MI}=\frac{H_{\max}-H(P)}{H_{\max}}$$ (3)

Seven cross-frequency coupling types were examined: delta-theta, delta-low-beta, delta-high-beta, theta-low-alpha, theta-high-alpha, theta-low-beta, and theta-high-beta. Considering the functional characteristics of different cognitive stages, different time windows focused on different interregional PAC patterns. The N1 window primarily focused on coupling between frontal or fronto-central phase and occipital amplitude; the P2 window focused on coupling between frontal or fronto-central phase and parietal amplitude; the N2 window focused on local coupling within frontal and fronto-central regions; the P3 window focused on coupling between frontal or fronto-central phase and parietal amplitude; and the T0 window focused on full-period coupling between frontal or fronto-central phase and occipital or parietal amplitude.

#### dwPLI-based functional connectivity analysis

Functional connectivity analysis based on the debiased weighted phase lag index (dwPLI) was used to evaluate the stability of phase synchronization between brain regions. The dwPLI was used to quantify interregional functional connectivity strength (Vinck et al., 2011). Because dwPLI is based on weighted phase-lag information from the imaginary component of the cross-spectrum, it can reduce spurious connectivity caused by volume conduction and common reference effects. Debiasing further reduces the influence of sample size on estimation bias, thereby improving the reliability of cross-trial and between-group comparisons. The debiased estimator was computed using Equation 4, where Im(X) represents the imaginary part of the cross-spectrum.


dwPLI=∑t=1n∣Im(Xt)∣sgn(Im(Xt))∑t=1n∣Im(Xt)∣ (4)

Functional connectivity analysis was conducted across five frequency bands: theta (4–8 Hz), low alpha (8–10 Hz), high alpha (10–13 Hz), low beta (13–20 Hz), and high beta (20–30 Hz). The analysis examined five cognitive time windows, N1, P2, N2, P3, and T0, to describe dynamic network characteristics across task phases. In the spatial dimension, based on the four predefined core ROIs, six functional connectivity pairs were calculated: frontal–parietal, frontal-occipital, fronto-central-parietal, fronto-central-occipital, frontal-fronto-central, and parietal-occipital.

### Statistical analysis

Categorical variables were analyzed using the chi-square test. Clinical scale scores and behavioral variables were first tested for normality. For normally distributed data, homogeneity of variance was further examined before between-group comparisons. If homogeneity of variance was satisfied, independent-samples *t* tests were used; otherwise, Welch *t* tests were applied. For non-normally distributed data, Wilcoxon rank-sum tests were used. For all EEG features, including ERP, TF, PAC, and dwPLI features, between-group differences between the HN-MCI and NEN-MCI groups were assessed using two-sample permutation tests (Maris and Oostenveld, 2007), with 5,000 permutations and mean difference as the test statistic. Effect sizes were expressed as Cohen’s d. Between-group comparisons of demographic variables, clinical scale scores, MoCA indices, and behavioral task outcomes were reported as raw *p* values. For EEG features, including ERP, TF, PAC, and dwPLI features, multiple comparisons were controlled using the Benjamini-Hochberg false discovery rate (FDR) procedure, with statistical significance defined as *q* < 0.05.

### Software

EEG preprocessing, feature extraction, and statistical analyses were performed in Python (v3.13.5). The packages used included MNE, NumPy, SciPy, pandas, h5py, openpyxl, scikit-learn, XGBoost, SHAP, and Matplotlib. Functional connectivity topological maps and brain network visualizations were generated using BrainNet Viewer (v1.7), with coordinates based on standard Montreal Neurological Institute (MNI) coordinates derived from the international 10–20 system (Koessler et al., 2009).

### Machine learning

A total of 372 candidate features were extracted, covering five modalities: 12 clinical and behavioral features, 12 ERP features, 100 TF power features, 98 PAC features, and 150 dwPLI functional connectivity features. Clinical and behavioral features included MoCA domain scores, ACC, RT and IES. ERP features included the amplitudes and latencies of six ERP components. TF power features were extracted according to frequency bands, time windows, and ROIs. PAC features were derived from interregional phase-amplitude coupling indices in different time windows. dwPLI features were derived from interregional functional connectivity indices across frequency bands and time periods.

To reduce the multiple-comparison burden in the group-level statistical analyses, statistical comparisons were organized within predefined neurophysiological feature families rather than across an unrestricted exploratory feature space. ERP comparisons were corrected across the ERP feature set. Time-frequency power and dwPLI comparisons were corrected within frequency-band × time-window families, and PAC comparisons were corrected within coupling-type × time-window families using Benjamini-Hochberg FDR correction. These feature families were defined *a priori* according to task-related processing stages, canonical EEG frequency bands, ERP components, and predefined ROI or ROI-pair structures.

Clinical scales, behavioral data, and multidimensional electrophysiological indices (ERP, TF, PAC, and dwPLI) were integrated to construct the initial feature set. Feature selection, scaling, redundancy removal, and hyperparameter tuning were all performed strictly within the training folds to avoid information leakage. First, only neurophysiological features that reached significance in the training set according to two-sample permutation tests followed by grouped FDR correction (*q* < 0.05) were retained. Pearson correlation analysis was then performed on the retained features. When the absolute correlation coefficient between any two features was |*r*| > 0.7, they were considered redundant, and only the feature with the larger absolute Cohen’s d in the current training set was retained to reduce collinearity and improve feature independence. All continuous features were z-scored within the training folds, and the parameters derived from the training set were applied to the corresponding test set to prevent information leakage. After training-fold feature screening, redundancy reduction, and model evaluation, a low-dimensional set of nine multimodal features was retained for the final stacking model and SHAP-based interpretation.

The stacking decision process can be expressed as Equation 5:


$$\overset{\wedge }{y}=\text{Meta}(\sum_{i=1}^{k}w_{i}\cdot P_{i}(x))$$ (5)

where $P_{i}(x)$ denotes the predicted probability generated by the i-th base learner, $w_{i}$ denotes the weight assigned by the meta-learner, and the summation is taken over all base learners.

The classification model used a two-layer stacking ensemble framework. The base learners included SVM with a radial basis function kernel (SVM-RBF), linear SVM (SVM-Linear), ridge logistic regression, L2-regularized logistic regression, XGBoost, and random forest. Ridge logistic regression was used as the meta-learner. To reduce overfitting, out-of-fold predictions from the base learners were generated through seven-fold internal cross-validation, and hyperparameters were optimized using GridSearchCV. Final predictions were obtained by the meta-learner through fusion of the predicted probabilities output by the base learners.

Model performance was evaluated using 10 repeats of five-fold stratified cross-validation. In each round, stratified bootstrap resampling (*n* = 100) was further performed on the outer test set to assess the stability of performance metrics. Evaluation metrics included AUC, accuracy, sensitivity, specificity, and F1 score. Each statistic was presented as the mean and standard deviation derived from repeated cross-validation. Label permutation testing was conducted to assess whether model performance was significantly superior to chance. Maintaining a consistent feature matrix, group labels were randomly permuted, and the comprehensive feature selection, model training, and cross-validation process was executed for 1,000 iterations to get the null AUC distribution. The empirical *p*-value was defined as the ratio of permuted AUC values that are greater than or equal to the observed AUC.

SHAP was employed to compute Shapley values for the chosen features, thereby elucidating the extent and direction of each feature’s influence on the classification decisions of the final stacking model.

The Shapley value for feature i was computed in accordance with Equation 6:


$$\phi_{i}=\sum_{S\subseteq F\backslash \{i\}}\frac{|S|!(|F|-|S|-1)!}{|F|!}[f(S\cup \{i\})-f(S)]$$ (6)

Let F represent the complete feature set, S signify a subset of features omitting feature *i*, and *f*(*S*) imply the output function of the model.

To characterize the multicollinearity structure among the final selected features after redundancy removal, Pearson correlation analysis was performed on the nine features included in the final stacking model, and the results were visualized using a correlation ellipse heatmap. Two-sided associations with *p* < 0.05 were marked with black borders.

## Results

### General characteristics of the study participants

**Figure 4:**
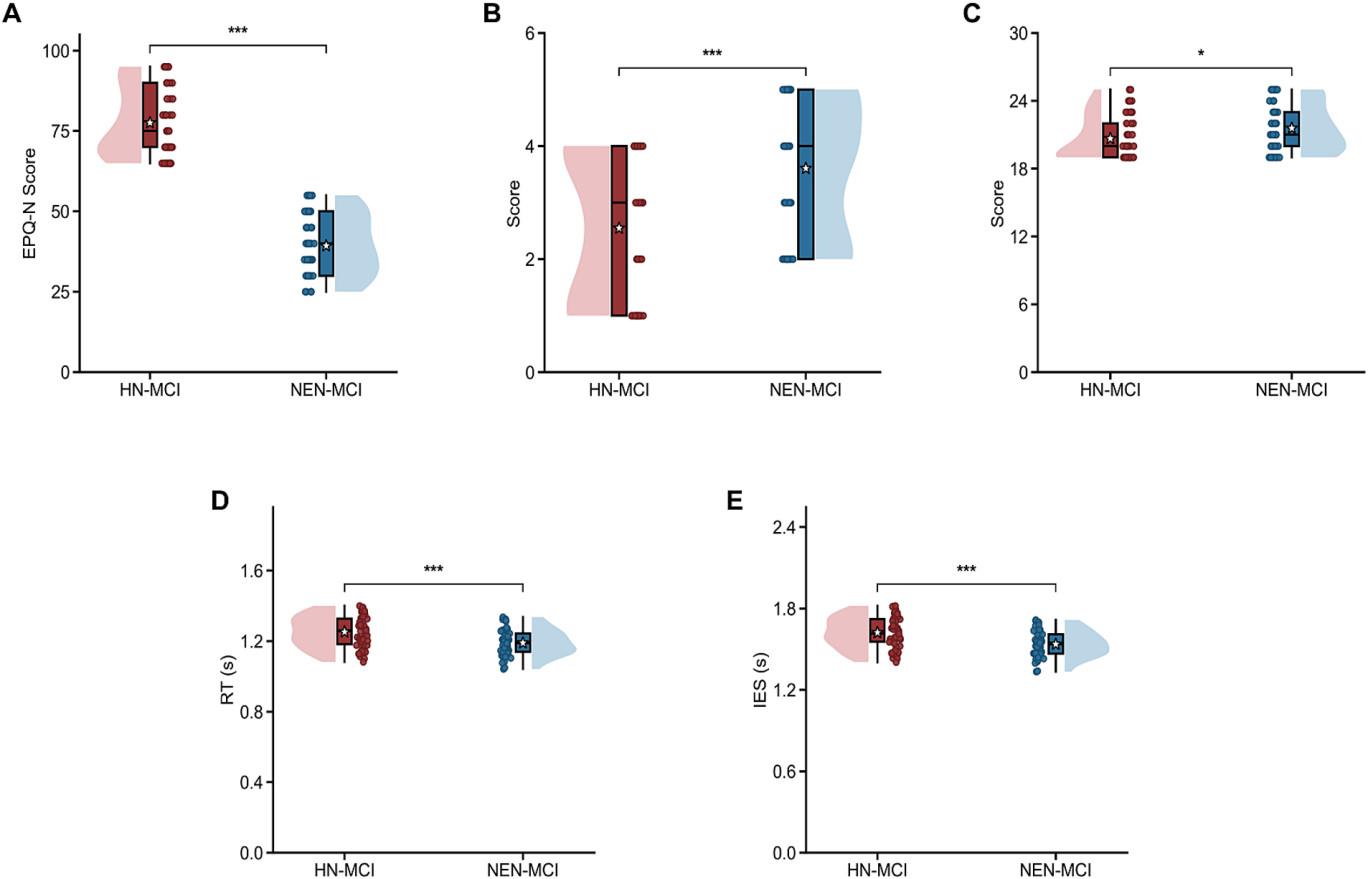
Between-group comparison of neuropsychological and behavioral indicators between HN-MCI and NEN-MCI. **(A)** EPQ-N scores were higher in the HN-MCI group than in the NEN-MCI group. **(B)** MoCA visuospatial/executive scores were lower in the HN-MCI group than in the NEN-MCI group. **(C)** Corrected MoCA total scores were lower in the HN-MCI group than in the NEN-MCI group. **(D)** Reaction times were longer in the HN-MCI group than in the NEN-MCI group. **(E)** Inverse efficiency scores (IES) were higher in the HN-MCI group than in the NEN-MCI group. Red indicates the HN-MCI group, and blue indicates the NEN-MCI group. Asterisks indicate statistically significant between-group differences (*p < 0.05, ***p < 0.001).

The two groups were matched for sex, age, and years of education. No significant between-group differences were observed in sex distribution, age, or years of education. Both the HN-MCI and NEN-MCI groups included 25 men and 28 women (*χ^2^* = 0.000, *p* = 1.000). The mean age was 54.75 ± 4.93 years in the HN-MCI group and 54.64 ± 4.97 years in the NEN-MCI group (*t* = 0.118, *p* = 0.907). Years of education were also comparable between groups (11.13 ± 1.18 vs. 11.32 ± 1.09 years, *Z* = −0.670, *p* = 0.503). As expected, the HN-MCI group had significantly higher EPQ-N scores than the NEN-MCI group (77.55 ± 10.95 vs. 39.25 ± 9.53, *Z* = 8.875, *p* < 0.001; Figure 4A; Supplementary Table S2). Within both the HN-MCI and NEN-MCI groups, EEG-included and EEG-not-included participants did not differ significantly in sex, age, education, EPQ-N, MoCA total score, or MoCA visuospatial/executive score (all *p* > 0.05; Supplementary Table S1).

### Neuropsychological and behavioral performance

For MoCA performance, the HN-MCI group showed significantly lower visuospatial/executive scores than the NEN-MCI group (2.55 ± 1.31 vs. 3.60 ± 1.20, *Z* = −3.949, *p* < 0.001; Figure 4B). The corrected MoCA total score was also significantly lower in the HN-MCI group than in the NEN-MCI group (20.66 ± 1.91 vs. 21.57 ± 2.08, *Z* = −2.430, *p* = 0.015; Figure 4C; Supplementary Table S2). No significant between-group differences were observed in the remaining MoCA domains, including naming, attention, language, abstraction, delayed recall, and orientation (all *p* > 0.05; Supplementary Table S2).

During the modified visuospatial working memory task, accuracy did not differ significantly between groups (0.772 ± 0.008 vs. 0.774 ± 0.007, *t* = −1.425, *p* = 0.157). However, the HN-MCI group showed significantly longer reaction times than the NEN-MCI group (1.252 ± 0.088 s vs. 1.189 ± 0.073 s, *t* = 4.018, *p* < 0.001; Figure 4D) and significantly higher inverse efficiency scores (1.623 ± 0.113 s vs. 1.537 ± 0.094 s, *t* = 4.237, *p* < 0.001; Figure 4E; Supplementary Table S2).

### ERP differences between HN-MCI and NEN-MCI

**Figure 5:**
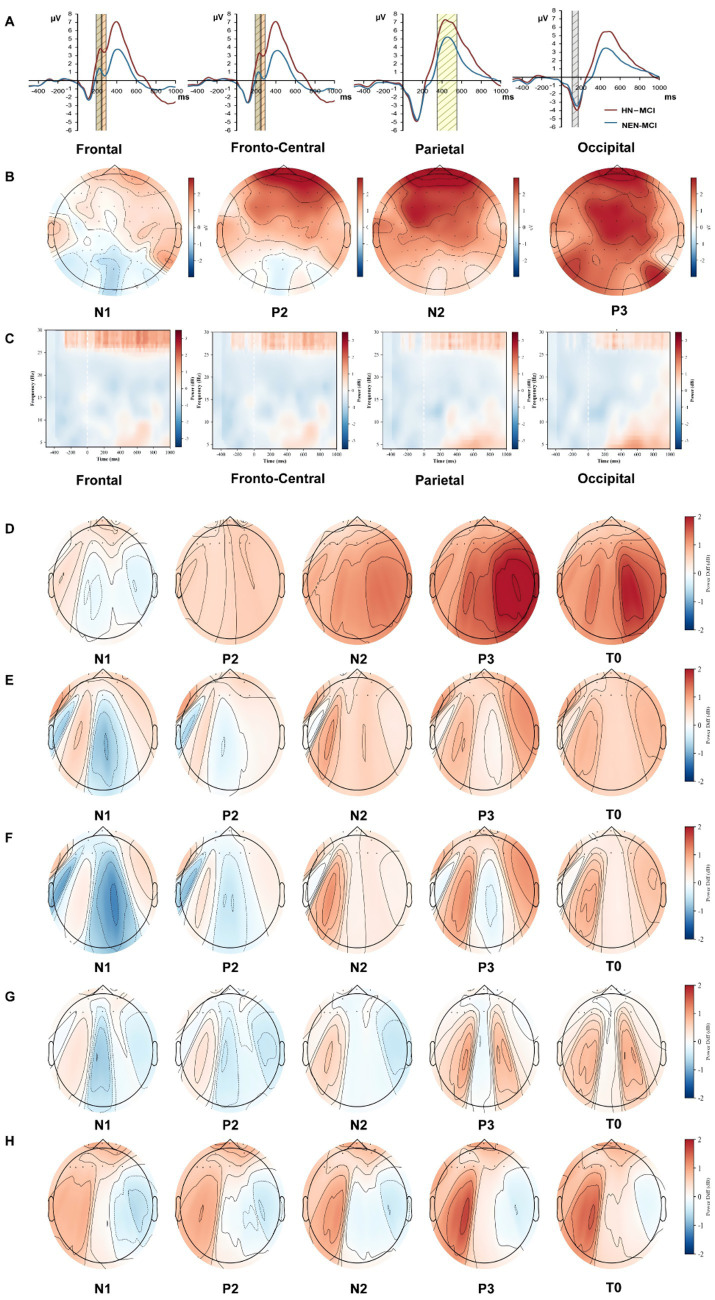
Differences in ERP, time-frequency power, and scalp topography between HN-MCI and NEN-MCI. **(A)** Average ERP waveforms for HN-MCI (red) and NEN-MCI (blue) across frontal, fronto-central, parietal, and occipital ROIs; shaded areas mark N1, P2, N2, and P3 windows. **(B)** ERP scalp topographies of amplitude differences (HN-MCI minus NEN-MCI, μV). **(C)** Time-frequency power differences across 4-30 Hz in the four ROIs (dB). **(D-H)** Scalp topographies of theta, low-alpha, high-alpha, low-beta, and high-beta power differences. Warm and cool colors indicate higher power in HN-MCI and NEN-MCI, respectively.

Between-group comparisons of ERP components revealed no significant differences in N1 amplitude or latency in the occipital region (all *q* > 0.05). For the P2 component, the HN-MCI group showed significantly higher mean amplitudes than the NEN-MCI group in the frontal and fronto-central regions, with between-group differences of 2.29 μV and 2.14 μV, respectively (*q* = 0.013 and *q* = 0.024). No significant between-group differences were observed in P2 latency (all *q* > 0.05; Figures 5A,B).

For the N2 component, the HN-MCI group showed reduced N2 negativity in the frontal and fronto-central regions, with between-group differences of 2.64 μV and 2.60 μV, respectively (both *q* = 0.013). N2 latency did not differ significantly between groups (all *q* > 0.05; Figures 5A,B). For the P3 component, the HN-MCI group showed significantly higher parietal amplitude than the NEN-MCI group, with a between-group difference of 2.22 μV (*q* = 0.002). P3 latency did not differ significantly between groups (*q* > 0.05; Figures 5A,B; Supplementary Table S3).

Overall, the HN-MCI group showed a pattern of increased P2 amplitude, reduced N2 negativity, and increased P3 amplitude, whereas component latencies did not differ significantly between groups.

### Time-frequency power differences

Time-frequency analysis showed that significant between-group differences were mainly distributed in the theta band, with additional differences observed in the low-alpha and high-beta bands (Figure 5C). In the theta band, significant differences were observed across frontal, fronto-central, parietal, and occipital regions. In the frontal and fronto-central regions, significant differences appeared in the P3 window (both *q* = 0.019) and the T0 window (both *q* = 0.006). In the parietal and occipital regions, significant theta-band differences were observed in the N2, P3, and T0 windows, with the strongest effects in the T0 window (all *q* < 0.001; Figure 5D).

In the low-alpha band, significant differences were fewer and were mainly concentrated in the T0 window across frontal, fronto-central, parietal, and occipital regions (*q* = 0.023 for the frontal region; all *q* = 0.022 for the remaining regions; Figure 5E). No significant between-group differences were observed in the high-alpha or low-beta bands (all *q* > 0.05; Figures 5F,G). High-beta differences were observed as a secondary pattern and were mainly distributed in frontal, fronto-central, and parietal regions. All significant high-beta effects reflected higher power in the HN-MCI group than in the NEN-MCI group. In the frontal region, significant differences were observed across the N1, P2, N2, P3, and T0 windows. In the fronto-central and parietal regions, significant differences were observed in the N1, P2, and T0 windows. No significant high-beta differences were observed in the occipital region (all *q* > 0.05; Figure 5H; Supplementary Table S4).

### PAC and dwPLI-based functional connectivity differences

**Figure 6:**
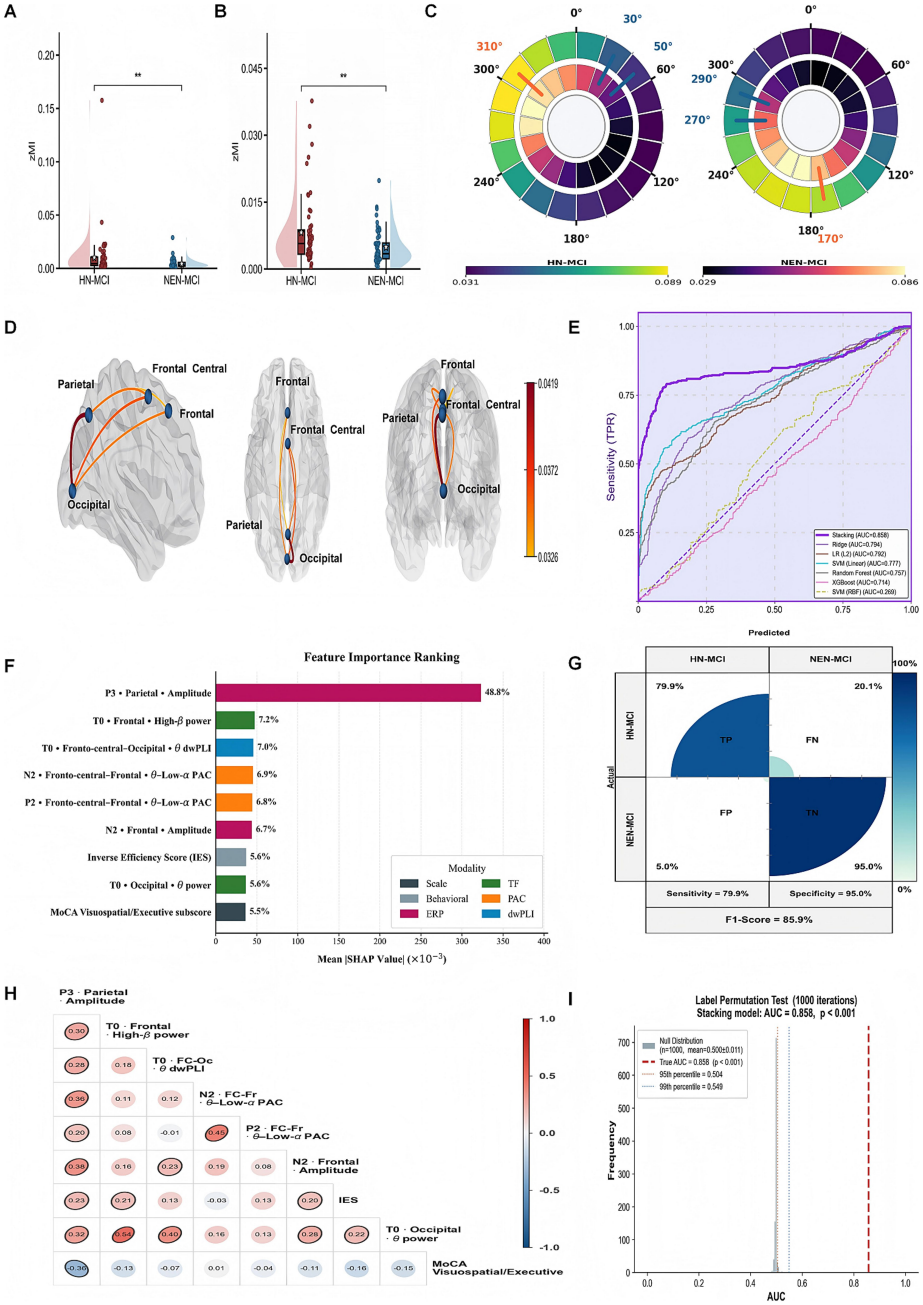
Discriminative PAC, functional connectivity, and multimodal machine learning classification in HN-MCI. **(A, B)** Theta-to-low-alpha PAC differences in the N2 and P2 windows. **(C)** Phase-amplitude distributions of significant PAC features; outer and inner rings denote HN-MCI and NEN-MCI, respectively. **(D)** Theta-band dwPLI connectivity differences in the T0 window. **(E)** ROC curves from repeated stratified five-fold cross-validation. **(F)** SHAP feature contributions for the stacking model. **(G)** Confusion matrix. **(H)** Pearson correlation ellipse heatmap; black borders indicate significant correlations (*p* < 0.05). **(I)** Label permutation test showing null and observed AUCs (permutation *p* < 0.001).

PAC analysis showed significant between-group differences only in a limited set of coupling patterns. Specifically, under the condition of fronto-central phase and frontal amplitude coupling, theta-to-low-alpha PAC differed significantly between groups in both the P2 window (*q* = 0.025) and the N2 window (*q* = 0.015). In both cases, PAC values were higher in the HN-MCI group than in the NEN-MCI group (Figures 6A–C). No significant differences were observed in the remaining phase-amplitude frequency pairs or time windows (all *q* > 0.05; Supplementary Table S5).

Functional connectivity analysis based on dwPLI revealed significant between-group differences only in a small number of ROI pairs, all of which were concentrated in the theta band during the T0 window. Specifically, significant differences were observed between the fronto-central and occipital regions (*q* = 0.021), fronto-central and parietal regions (*q* = 0.029), frontal and occipital regions (*q* = 0.021), frontal and parietal regions (*q* = 0.035), and parietal and occipital regions (*q* = 0.029). All significant theta-band T0 connections were stronger in the HN-MCI group than in the NEN-MCI group (Figure 6D). No significant differences were observed in other frequency bands or time windows (all *q* > 0.05; Supplementary Table S6).

### Multimodal machine learning classification and SHAP interpretability analysis

Classification models were constructed using clinical scale scores, behavioral measures, and multidimensional electrophysiological features, including ERP, TF, PAC, and dwPLI features. Based on the fold-wise AUCs derived from the saved out-of-fold prediction probability files used for the ROC curves, L2-regularized logistic regression, ridge logistic regression, and SVM-Linear showed the best base-learner performance, with AUC values of 0.792 ± 0.094, 0.794 ± 0.094, and 0.777 ± 0.096, respectively. Random forest and XGBoost showed moderate performance (AUC = 0.757 ± 0.103 and 0.714 ± 0.102), whereas SVM-RBF showed the lowest performance among the base learners (AUC = 0.269 ± 0.189). The stacking model achieved the highest overall AUC (0.858 ± 0.091; Table 1).

**Table 1:** Classification performance of base classifiers and the stacking ensemble model.

Model	AUC
LR (L2)	0.792 ± 0.094
Ridge	0.794 ± 0.094
SVM (Linear)	0.777 ± 0.096
Random Forest	0.757 ± 0.103
XGBoost	0.714 ± 0.102
SVM (RBF)	0.269 ± 0.189
Stacking	0.858 ± 0.091

Additional classification metrics for the stacking model are reported in Table 2. Base-learner AUCs were calculated from the saved out-of-fold prediction probability files used to draw the ROC curves, ensuring consistency between Table 1 and Figure 6E. SVM-RBF showed the lowest performance among the base learners in the present repeated cross-validation setting (AUC = 0.269 ± 0.189), which may be related to the small-sample and high-dimensional nature of the data. This result is reported for completeness.

The final stacking model showed good overall classification performance across 10 repeats of five-fold stratified cross-validation, with an AUC of 0.858 ± 0.091, accuracy of 0.825 ± 0.070, sensitivity of 0.712 ± 0.138, specificity of 0.938 ± 0.082, F1 score of 0.796 ± 0.093, and Brier score of 0.150 ± 0.040 (Figure 6E,G; Table 2). Permutation testing showed that the null AUC distribution was centered near chance level (0.500 ± 0.011), whereas the observed model AUC (0.858) was clearly separated from the null distribution (permutation *p* < 0.001; Figure 6I).

**Table 2:** Classification performance of the final stacking ensemble model.

Metric	Mean ± SD
AUC	0.858 ± 0.091
Accuracy	0.825 ± 0.070
Sensitivity	0.712 ± 0.138
Specificity	0.938 ± 0.082
F1 score	0.796 ± 0.093
Brier score	0.150 ± 0.040

The stacking model integrates six base classifiers (Ridge, LR-L2, SVM-Linear, SVM-RBF, XGBoost, Random Forest) via a logistic regression meta-learner. The optimal classification threshold was determined per fold by maximizing the Youden index on the training ROC curve. AUC labels in the ROC figure are rounded to three decimal places; three-decimal values are reported here for precision. Brier score measures probabilistic calibration error (lower = better; perfect = 0).

SHAP analysis was used to interpret feature contributions in the final stacking model. The ERP feature P3 parietal amplitude showed the largest contribution (323.541 × 10^−3^, 48.83%). The next most important features were T0 frontal high-beta power (47.628 × 10^−3^, 7.19%), T0 fronto-central-occipital theta dwPLI (46.069 × 10^−3^, 6.95%), N2 fronto-central-frontal theta-to-low-alpha PAC (45.556 × 10^−3^, 6.88%), P2 fronto-central-frontal theta-to-low-alpha PAC (44.726 × 10^−3^, 6.75%), and N2 frontal amplitude (44.293 × 10^−3^, 6.69%). Behavioral IES (37.312 × 10^−3^, 5.63%), T0 occipital theta power (36.888 × 10^−3^, 5.57%), and the MoCA visuospatial/executive subscore (36.529 × 10^−3^, 5.51%) also contributed to model classification (Figure 6F; Supplementary Table S7).

Pearson correlation analysis of the nine final selected features identified 16 significant pairwise associations (*p* < 0.05; Figure 6H; Supplementary Table S8). The strongest positive association was observed between T0 frontal high-beta power and T0 occipital theta power (*r* = 0.539, *p* < 0.001). Other moderate positive associations included the correlation between N2 and P2 fronto-central-frontal theta-to-low-alpha PAC features (*r* = 0.455, *p* < 0.001), T0 fronto-central-occipital theta dwPLI and T0 occipital theta power (*r* = 0.396, *p* < 0.001), P3 parietal amplitude and N2 frontal amplitude (*r* = 0.384, *p* < 0.001), and P3 parietal amplitude and N2 fronto-central-frontal theta-to-low-alpha PAC (*r* = 0.361, *p* < 0.001). The MoCA visuospatial/executive subscore showed a significant negative correlation with parietal P3 amplitude (*r* = −0.362, *p* < 0.001). IES showed weak but significant positive correlations with P3 parietal amplitude (*r* = 0.225, *p* = 0.020), T0 occipital theta power (*r* = 0.224, *p* = 0.021), T0 frontal high-beta power (*r* = 0.211, *p* = 0.030), and N2 frontal amplitude (*r* = 0.202, *p* = 0.038). These weak-to-moderate associations indicate that the final selected features retained complementary rather than highly redundant multimodal information.

## Discussion

The present study systematically compared multimodal neurodynamic differences between individuals with HN-MCI and those with NEN-MCI during a visuospatial working memory task. The HN-MCI group showed abnormalities across ERP, oscillatory activity, cross-frequency coupling, and large-scale functional connectivity, together with lower corrected MoCA total and visuospatial/executive scores and reduced behavioral efficiency. Behaviorally, individuals with HN-MCI maintained comparable task accuracy but showed longer reaction times and higher inverse efficiency scores, suggesting greater cognitive cost during working memory processing. The stacking ensemble model provided effective multimodal classification, and SHAP analysis indicated that clinical, behavioral, ERP, time-frequency, PAC, and functional connectivity features all contributed to classification. These findings suggest that HN-MCI-related abnormalities are not confined to a single measure, but reflect coordinated changes across behavioral performance and multiple layers of brain activity.

Compared with the NEN-MCI group, the HN-MCI group showed increased P2 amplitude, reduced N2 negativity, and increased P3 amplitude. The P2 component is closely related to early attentional allocation (Potts, 2004), and the increased P2 amplitude in HN-MCI may indicate that more cognitive resources are required at the initial stage of task processing to maintain attentional focus. This discovery is in line with ERP findings from working memory tasks in normal individuals with high levels of neuroticism (Xiu et al., 2022). Attentional control theory claims the internal emotional interference related to neuroticism may exhaust attentional resources at the beginning of tasks (Eysenck et al., 2007). The N2 component is usually related to conflict monitoring and cognitive control (Folstein and Van Petten, 2008). Reduced N2 negativity in the HN-MCI group, especially after increased early P2 engagement, may reflect insufficient resources available for sustained monitoring and interference suppression. A comparable decrease in N2 amplitude has been documented in persons exhibiting high trait anxiety during cognitive interference (Dennis and Chen, 2009), corroborating the notion that neuroticism-associated negative affect may diminish cognitive control efficacy. The P3 component pertains to subsequent cognitive resource allocation and the update of working memory (Polich, 2007). The heightened parietal P3 amplitude in the HN-MCI cohort, coupled with maintained accuracy but protracted reaction times, indicates compensatory late-stage resource allocation rather than enhanced processing efficiency (Correa-Jaraba et al., 2018). The ERP pattern of P2 over-engagement, N2 control weakening, and P3 compensatory increase suggests that heightened neuroticism may persistently influence the neuronal temporal dynamics of working memory processing in MCI.

The HN-MCI group showed a persistent high-load pattern at the levels of neural oscillation and brain network coordination, consistent with the ERP results. Task-related theta oscillations are closely related to the maintenance of working memory, cognitive control, and attentional regulation (van Engen et al., 2024; Wischnewski et al., 2024). The present study found that theta-band abnormalities ranged from increased local power to enhanced dwPLI-based functional connectivity. The HN-MCI group showed elevated theta activity in frontal, fronto-central, parietal, and occipital regions, as well as enhanced theta-band connectivity among these regions across the full task period. Because dwPLI indexes phase-lagged interregional synchronization while reducing spurious connectivity from volume conduction, these findings suggest that individuals with HN-MCI may rely on broader and more sustained large-scale network coordination to maintain visuospatial working memory performance. The restriction of significant connectivity differences to the T0 full-task window, rather than ERP-defined time windows, suggests that the connectivity abnormality in HN-MCI may reflect a tonic network state rather than a transient response to specific stimulus events. In addition, the HN-MCI group showed sustained increases in high-beta activity, mainly in frontal and parietal regions, across multiple time periods. High-beta activity is associated with sustained alertness, emotional load, and ongoing cognitive effort (Park et al., 2026), indicating that elevated neuroticism may be associated with prolonged mental arousal and increased resource use. At the cross-frequency level, the HN-MCI group showed greater theta-to-low-alpha coupling between fronto-central phase and frontal amplitude. Theta activity is thought to support the maintenance of working memory content, whereas low-alpha activity is involved in inhibitory filtering of task-irrelevant visual stimuli (Palacios-García et al., 2021; Wischnewski et al., 2024). Greater theta-to-low-alpha coupling may therefore indicate that individuals with HN-MCI require increased cross-frequency coordination to maintain task-relevant information and suppress irrelevant interference. This pattern is consistent with studies showing increased theta activity and altered alpha modulation under stress- and anxiety-related conditions (Jaiswal et al., 2019; Palacios-García et al., 2021). Collectively, augmented theta activity, persistent high-beta activity, elevated theta-to-low-alpha coupling, and reinforced theta-band dwPLI connectivity suggest that individuals with HN-MCI may require extensive oscillatory engagement and network synchronization to sustain task performance, potentially contributing to reduced behavioral processing efficiency.

The previously described multilayer neurodynamic abnormalities were also supported by cognitive and behavioral evidence. The HN-MCI group showed significantly lower corrected MoCA total scores and lower MoCA visuospatial/executive scores than the NEN-MCI group, whereas the remaining MoCA domains showed no significant differences. Large-sample longitudinal studies have shown that higher neuroticism and associated trait anxiety are related to executive dysfunction and lower visuospatial abilities in older individuals (Chapman et al., 2017; Cerino et al., 2020), and the current findings align with this perspective. At the behavioral level, the HN-MCI group displayed significantly longer reaction times and higher inverse efficiency scores, whereas accuracy remained comparable between groups. This pattern indicates that individuals with HN-MCI can maintain task accuracy, but at a higher cognitive cost. The dissociation between prolonged response times and maintained accuracy aligns with a previous meta-analysis (Moran, 2016) and findings from trait anxiety studies (Basten et al., 2011), suggesting that emotional burden may first impair processing efficiency rather than directly reduce performance accuracy. These behavioral findings provide functional support for the high-cost and low-efficiency neural processing pattern indicated by the electrophysiological results.

This study applied a stacking ensemble framework to integrate clinical scale scores, behavioral indicators, and EEG features from ERP, time-frequency analysis, PAC, and dwPLI. The final stacking model showed good classification performance in distinguishing HN-MCI from NEN-MCI, with an AUC of 0.858, and outperformed the individual base learners. This finding supports the value of multimodal feature fusion for capturing heterogeneous neurodynamic alterations in MCI. Previous studies have similarly shown that combining EEG-derived features or integrating neuroimaging and clinical information can improve the classification of MCI and Alzheimer’s disease (Ieracitano et al., 2020; Henríquez and Araya, 2024; Mlinarič et al., 2026). In line with these studies, the present results suggest that ERP timing, oscillatory activity, cross-frequency coordination, and large-scale functional connectivity provide complementary information for characterizing neuroticism-related neurodynamic heterogeneity in MCI.

SHAP-based interpretability analysis further clarified which multimodal features contributed most to the stacking classification decision. Among the nine final selected features, parietal P3 amplitude was the dominant contributor, accounting for 48.83% of the total mean absolute SHAP contribution. This finding suggests that late-stage cognitive resource allocation is a central discriminator between HN-MCI and NEN-MCI. The parietal cortex has been implicated in working memory updating, top-down attentional allocation, and emotional regulation (Menon, 2011; Servaas et al., 2013; Goodkind et al., 2015), which supports the interpretation that elevated neuroticism in MCI may be linked to altered late-stage cognitive resource processing. In addition to P3 amplitude, the retained features included frontal high-beta power, theta-band dwPLI connectivity, N2- and P2-window theta-to-low-alpha PAC features, N2 frontal amplitude, behavioral IES, occipital theta power, and the MoCA visuospatial/executive subscore. This pattern indicates that the classification model did not rely on a single marker, but integrated information across temporal, spectral, cross-frequency, network, behavioral, and cognitive domains. Pearson correlation analysis of the final selected features identified 16 significant pairwise associations, most of which were weak to moderate in magnitude. The MoCA visuospatial/executive subscore was negatively correlated with parietal P3 amplitude (*r* = −0.362, *p* < 0.001), linking poorer visuospatial/executive performance with stronger late-stage electrophysiological resource allocation. These weak-to-moderate associations suggest that the retained features preserved complementary rather than highly redundant multimodal information. Together with the label permutation results, this multimodal feature profile provides neurobehaviorally meaningful evidence for neurodynamic heterogeneity associated with HN-MCI.

The current findings indicate that high-neuroticism-related MCI may be characterized by excessive cognitive resource use and inefficient cognitive regulation during visuospatial working memory processing. This pattern appears to involve coordinated alterations in temporal processing, oscillatory activity, cross-frequency coupling, and large-scale functional connectivity, rather than a single electrophysiological abnormality. Personality-based stratification combined with multimodal EEG may therefore offer a useful approach for characterizing neurodynamic heterogeneity within MCI and identifying electrophysiological features relevant to individualized risk assessment.

Several limitations should be acknowledged. First, this study classified MCI participants into HN-MCI and NEN-MCI groups according to EPQ-N scores, but did not further examine the continuous effect of different neuroticism levels on MCI-related neurodynamics. The cross-sectional design also limits causal inference and cannot determine the long-term relationship between elevated neuroticism and MCI progression. Although participants with clear histories of major depression, anxiety disorders, schizophrenia, and other severe psychiatric conditions were excluded through medical history review, medication history, participant self-report, and clinical interview, standardized psychiatric diagnostic interviews and symptom scales for depression and anxiety were not administered. Therefore, residual confounding from subclinical depressive or anxiety symptoms cannot be fully excluded. Given the close relationship among neuroticism, anxiety, depression, and cognitive performance, future studies should incorporate systematic psychiatric assessments and statistically control current emotional symptoms when examining neuroticism-related EEG neurodynamic alterations in MCI. Longitudinal studies with finer-grained neuroticism stratification are also needed to determine whether the key electrophysiological features identified here can predict long-term cognitive decline. Second, although the comparison between EEG-included and EEG-not-included eligible participants showed no significant differences in the available demographic, EPQ-N, and cognitive variables, potential selection bias related to willingness to undergo EEG recording, behavioral-task completion, and EEG data quality control cannot be fully excluded. Third, scalp EEG has limited spatial resolution and cannot precisely localize deep limbic structures such as the amygdala and hippocampus, which are closely involved in neuroticism-related emotional regulation. Future studies combining EEG with fMRI may help verify the anatomical basis of cross-frequency coupling and network synchronization abnormalities at a higher spatial resolution, and peripheral physiological indicators such as heart rate variability and cortisol may provide additional information for linking central and peripheral stress-related regulation. Fourth, the generalizability of the stacking model has not yet been validated in an independent external cohort. Future multicenter studies across different EEG systems and broader populations are needed to further test the generalizability and stability of the present findings.

## Data Availability

The original contributions presented in the study are included in the article/Supplementary material, further inquiries can be directed to the corresponding author.
